# Supplementary material for: Tunable quantum criticalities in an isospin extended Hubbard model simulator
Source: Nature. 2022 Sep 14;609(7927):479–84. doi: 10.1038/s41586-022-05106-0 (PMC9477744; doi:10.1038/s41586-022-05106-0)
Supplement: Supplementary file 1 — This file contains Supplementary sections 1–15 and the Supplementary Figures. [file 41586_2022_5106_MOESM1_ESM.pdf]

nature portfolio

https://doi.org/10.1038/s41586-022-05106-0

Supplementary information

Tunable quantum criticalities in an isospin extended Hubbard model simulator

In the format provided by the authors and unedited

Supplementary Information

Tunable quantum criticalities in an isospin extended Hubbard model simulator

Qiao Li[1], Bin Cheng[2*], Moyu Chen[1], Bo Xie[3], Yongqin Xie[1], Pengfei Wang[1], Fanqiang Chen[1], Zenglin Liu[1], Kenji Watanabe[4], Takashi Taniguchi[5], Shi-Jun Liang[1], Da Wang[1], Chenjie Wang[6], Qiang-Hua Wang[1], Jianpeng Liu[3], Feng Miao[1*]

[1] National Laboratory of Solid State Microstructures, School of Physics, Institute of Brain-Inspired Intelligence, Collaborative Innovation Center of Advanced Microstructures, Nanjing University, Nanjing, China.

[2] Institute of Interdisciplinary Physical Sciences, School of Science, Nanjing University of Science and Technology, Nanjing 210094, China.

[3] School of Physical Science and Technology, ShanghaiTech laboratory for topological physics, ShanghaiTech University, Shanghai 200031, China.

[4] Research Center for Functional Materials, National Institute for Materials Science, 1-1 Namiki, Tsukuba 305-0044, Japan.

[5] International Center for Materials Nanoarchitectonics, National Institute for Materials Science, 1-1 Namiki, Tsukuba 305-0044, Japan.

[6] Department of Physics and HKU-UCAS Joint Institute for Theoretical and Computational Physics at Hong Kong, The University of Hong Kong, Pokfulam Road, Hong Kong, China

*Correspondence Email: bincheng@njust.edu.cn; miao@nju.edu.cn

I. Landau fan diagram

II. Thermal activation behaviour of charge neutrality gap (CNP)

III. Carrier density and displacement field dependence of Hall resistance

IV. Displacement field dependence of α_n

V. Characterizations of $n = 7n_0$ insulating state

VI. Mean field calculation

VII. Lower temperature data

VIII. Transport Data and theoretical analysis for positive D

IX. More device for generalized Wigner crystal

X. Estimation of the angle inhomogeneity

XI. Estimation of Joule heating

XII. Calculations of more fermi surface and r_s

XIII. Comparison of the resistance at moiré gap and Wigner crystal

XIV. Extraction of gaps at Wigner crystals

XV. Ruling out other mechanisms

I. Landau fan diagram

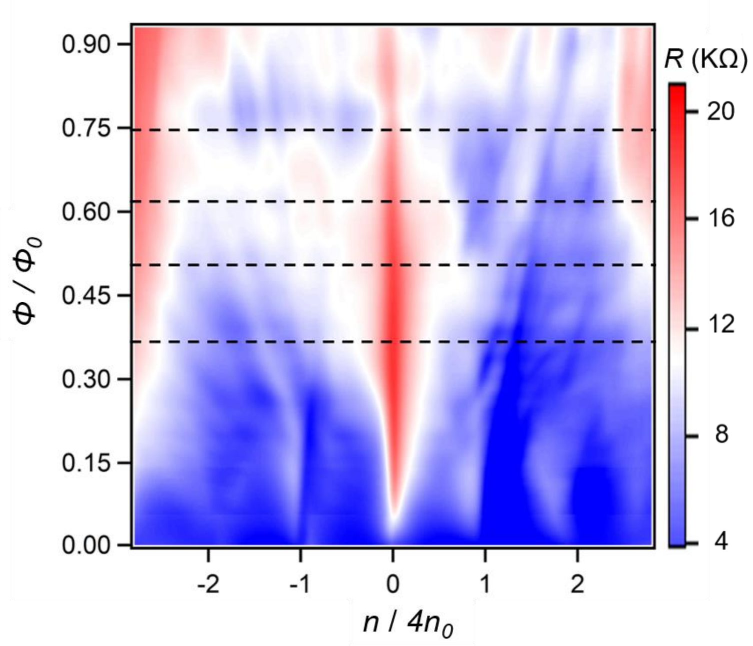

Figure S1. Landau fan diagram of cTDBG device measured at D=0 V/nm. Horizontal axis is the carrier density normalized by $4n_0$, and longitudinal axis is the vertical magnetic field normalized by quantum flux. At low magnetic field, several landau levels originate from moiré gaps; at high magnetic field, Hofstadter butterfly patterns appear. The black dashed lines from top to bottom represent the fractional quantum flux: $3/4\Phi_0$, $5/8\Phi_0$, $1/2\Phi_0$, $3/8\Phi_0$.

II. Thermal activation behaviour of charge neutrality gap (CNP)

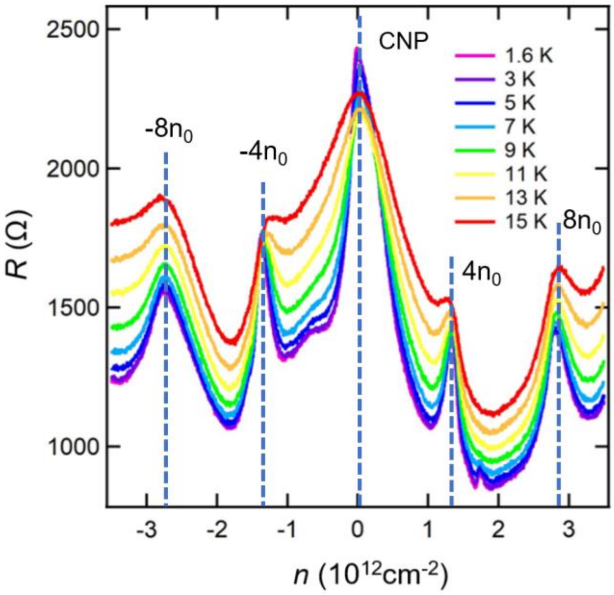

Figure S2. Carrier density n dependence of the longitudinal resistance at different temperatures (at D=0 V/nm). As temperature increases, the resistance at CNP decreases, while the resistances of the first and second moiré gap increase.

III. Carrier density and displacement field dependence of Hall resistance

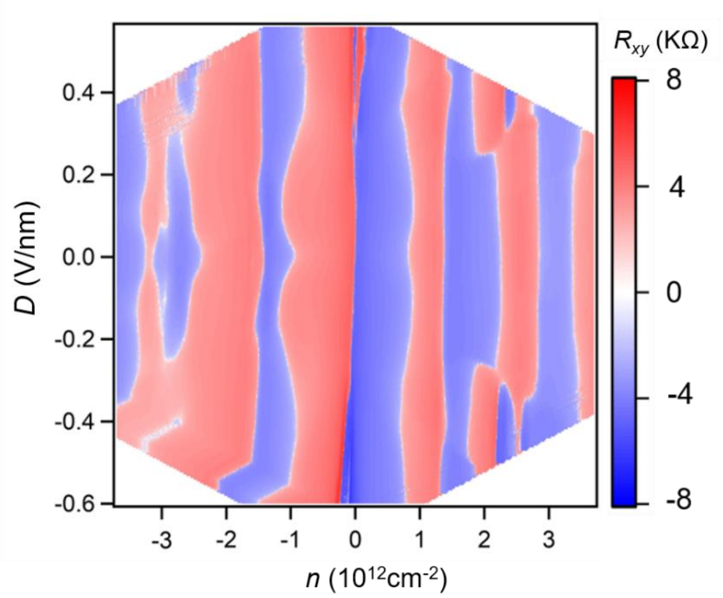

Figure S3. The mapping of longitudinal resistance as a function of carrier density and displacement field. R_{xy} changes sign abruptly at CNP and moiré gaps, which means the type of carrier density n changes.

IV. Displacement field dependence of α_n

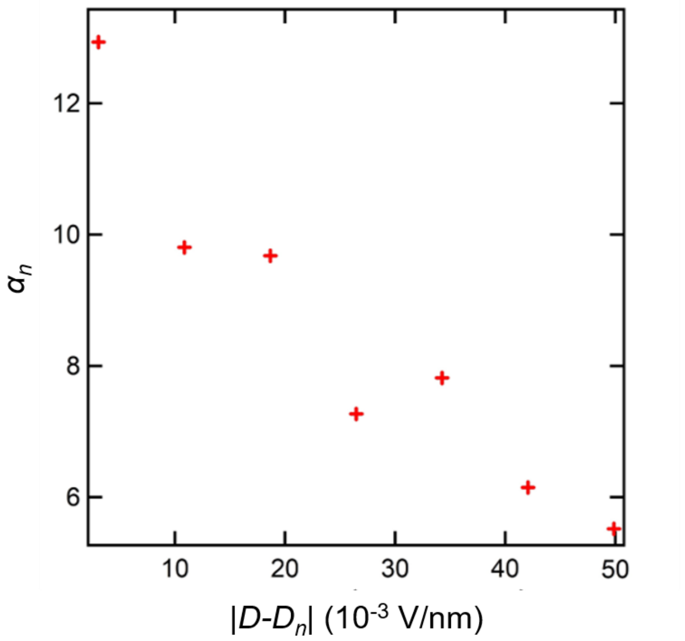

Figure S4. The coefficient α_n as a function of *D* near the normal metal-strange metal boundary, with α_n obtained from the fitting $R = \alpha_n T^2 + R_0$. α_n diverges as *D* approaches the critical point D_n.

V. Characterizations of $n = 7n_0$ insulating state

Our observations strongly indicate that the insulating state at n/n_0=7 is also a Wigner crystal. To make a comparison between the data/analysis at n/n_0=7+2/3 and 7, we presented $R(B_{//})/R(B_0)$ vs. B//, gap vs. B// and R vs. T in the **Fig. S5 (a)-(c)**. It is apparent that the n/n_0=7 state exhibits saturation of R in large $B_{//}$ and g-factor of larger than 2 as well as $R \sim T^{-1/2}$ relations, which are similar to those data/analysis in n/n_0=7+2/3 state. Remarkably, we have done data analysis for $7n_0$ insulating state, as shown in **Fig. S5(d)-(e)**, which is similar as the case in n/n_0=7+2/3 (Fig. 3b and 3c in the manuscript).

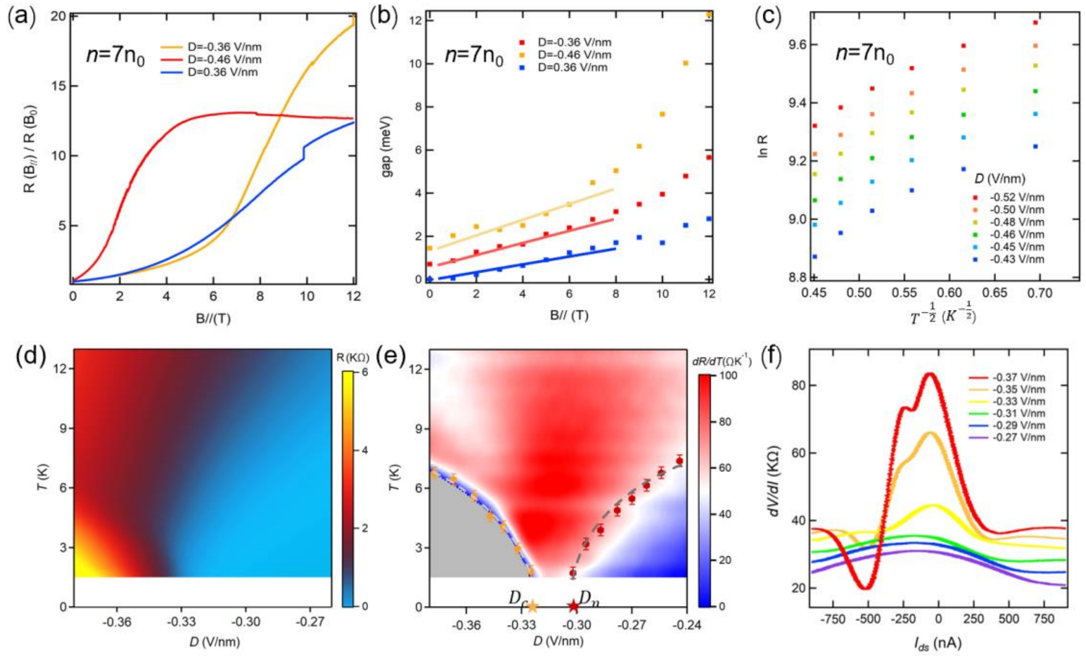

Figure S5: The characterization of the insulating peak at $7n_0$. (a) the normalized resistance vs parallel magnetic field at different *D*. (b) The extracted gap vs parallel magnetic field at three different *D* field for $n=7n_0$ peak, showing g-factor of around 9, 6, 4 extracted from the three different slopes. (c) The *R* in the log scale as a function of $T^{-1/2}$, showing an ES-type variable range hopping mechanism for electric transport at low temperature. (d) the 2D plot of resistance as a function of D and T. (e)The mapping of dR/dT as a function of D and T, showing similar quantum two-stage criticality as that in $(7+2/3)n_0$. (f) dV/dI in $n=7n_0$, showing prominent peak at zero I_{ds}. Notably, we find another asymmetric peak, which may indicate non-reciprocal charge transport is worthwhile to be investigated in the future.

VI. Mean field theory

we adopted a mean field method to solve SU(4) isospin extended Hubbard model on the triangular lattice. Here, we point out that such a strongly correlated model is difficult to solve exactly due to the lacking of a well-controlled many body technique especially in two dimensions till now. For example, Quantum Monte Carlo, as widely used in studying the SU(N) Hubbard model, suffers from significant negative sign problem in our case, tensor network related methods are also difficult to treat multi-component fermionic systems in more than one dimensions, and dynamical mean field theory (DMFT) cannot capture the spatial correlations unless using its cluster extensions. Therefore, here, we simply apply the mean field analysis in order to get some first insight of the dominant physics, as recently done by Das Sarma et al. (Phys. Rev. B 102, 201104; Phys. Rev. B 105, 041109) to study the Wigner-Mott transitions in a SU(2) case.

We start from the SU(4) extended Hubbard model on the triangular lattice:

$$H = -\sum_{\langle ij\rangle\alpha} t c_{i\alpha}^{\dagger} c_{j\alpha} - \sum_{i\alpha} \mu c_{i\alpha}^{\dagger} c_{i\alpha} + \sum_{i\alpha\neq\beta} U c_{i\alpha}^{\dagger} c_{i\alpha} c_{i\beta}^{\dagger} c_{i\beta} + \sum_{\langle ij\rangle\alpha\beta} V_{ij} c_{i\alpha}^{\dagger} c_{i\alpha} c_{j\beta}^{\dagger} c_{j\beta} \quad (1)$$

where $1 \leqslant \alpha, \beta \leqslant 4$. The chemical potential μ is to be tuned to achieve the averaged filling number 11/12 per flavor per site. U is the onsite Hubbard interaction and V_{ij} is the Coulomb interaction between neighbouring sites i and j. In our mean field analysis, we treat the Hubbard U only through the superexchange $J_{ij} = 4t^2_{ij}/U$, that means we neglect the U-term itself because in the mean field level it only causes magnetic orders (forbidden by Mermin-Wagner theorem) and is irrelevant to the experiment. Therefore, instead of Eq. 1, we turn to study its low energy effective t-J-V model

$$H = -\sum_{\langle ij\rangle\alpha} t c_{i\alpha}^{\dagger} c_{j\alpha} - \sum_{i\alpha} \mu c_{i\alpha}^{\dagger} c_{i\alpha} + \sum_{\langle ij\rangle\alpha\beta} \left(\frac{J_{ij}}{2} c_{i\alpha}^{\dagger} c_{i\beta} c_{j\beta}^{\dagger} c_{j\alpha} + V_{ij} c_{i\alpha}^{\dagger} c_{i\alpha} c_{j\beta}^{\dagger} c_{j\beta} \right) \quad (2)$$

In the mean field level, the interactions are decoupled through

$$\frac{J_{ij}}{2}c_{i\alpha}^{\dagger}c_{i\beta}c_{j\beta}^{\dagger}c_{j\alpha}=-\frac{J_{ij}}{2}\chi_{ij\alpha}c_{j\beta}^{\dagger}c_{i\beta}-\frac{J_{ij}}{2}c_{i\alpha}^{\dagger}c_{j\alpha}\chi_{ji\beta}+\frac{J_{ij}}{2}\chi_{ij\alpha}\chi_{ji\beta} \quad (3)$$

and

$$V_{ij}c_{i\alpha}^{\dagger}c_{i\alpha}c_{j\beta}^{\dagger}c_{j\beta}=V_{ij}n_{i\alpha}c_{j\beta}^{\dagger}c_{j\beta}+V_{ij}c_{i\alpha}^{\dagger}c_{i\alpha}n_{j\beta}-V_{ij}n_{i\alpha}n_{j\beta} \quad (4)$$

where $\chi_{ij\alpha} = \langle c^{\dagger}_{i\alpha}c_{j\alpha}\rangle$ and $n_{i\alpha} = \langle c^{\dagger}_{i\alpha}c_{i\alpha}\rangle$ are determined self-consistently by numerical calculations. In practice, we first perform calculations at small lattice size without restricting the structures of $n_{i\alpha}$ and $\chi_{ij\alpha}$. After finding the leading instabilities, we turn to larger lattice size by constructing super unit cells.

Typical mean field results are shown in **Fig. S6**. Roughly speaking, the nearest neighboring Coulomb interaction V_1 favors charge density wave (CDW): charge modulation with tripled moiré unit cell. On the other hand, the spin exchange J favors bond order or valence bond solid (VBS) (periodic modulation of χ). In our calculations, we find the nearest neighbor J_1 cannot induce VBS unless $J_1 \gg t_1$, which we believe is unphysical. But the next nearest neighbor J_2 is found to be able to induce a VBS by further breaking the translational symmetry on the basis of the CDW state, as shown in **Fig. S6 (d)**. If the above picture applies, the CDW and VBS are determined by V_1 and J_2, respectively, which however follow different tendencies towards increasing hopping. As $t_{1,2}$ increase by fixing their ratio t_2/t_1, V_1/t_1 decreases, leading to the vanishment of CDW, while J_2/t_1 increases leading to formation of the VBS near the CDW phase boundary. This may provide a possible explanation for the experimental observed two quantum critical points and the intermediate strange metal phase may be characterized by the (fluctuating) VBS, showing linear resistivity. Next, we add a Zeeman field B to represent the in-plane magnetic field. As $B_{//}$ increases, we find the VBS vanishes quickly, similar to the fate of the strange metal phase in the experiment.

We emphasize again that in mean field theory, both spatial and quantum fluctuations are neglected, which means that it cannot explain all the experimental results. But we can still get some qualitative understanding of the ground states and their dependence on $B_{//}$. Other numerical methods such as DMFT has been used in SU(2) extended Hubbard model on the square lattice with quarter filling (Kotliar et al., ***Nature***

Physics 4, 932–935 (2008)). In our SU(4) triangular lattice case, however, we may need to resort to the cluster extension of DMFT to involve both the CDW and VBS orderings, if they do exist, which is left for future work.

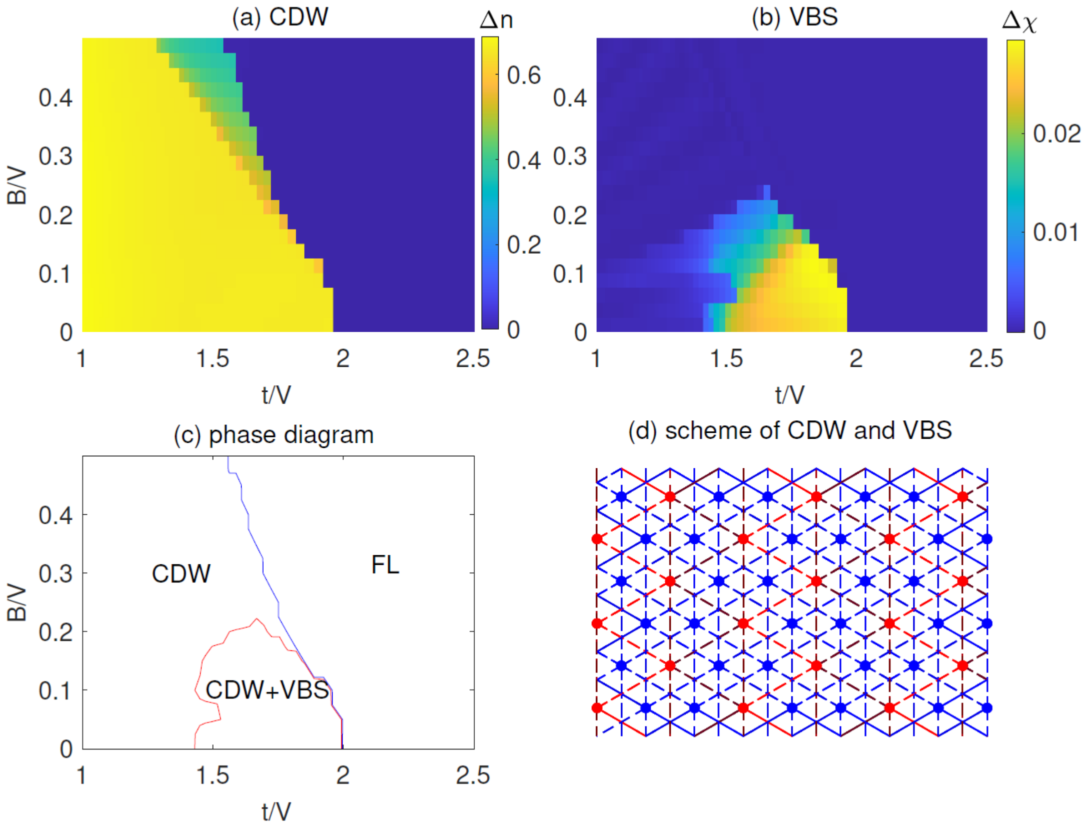

Figure S6: Typical mean field results at $J_2 = 4t_1^2/6V_1$. (a) The CDW order parameter, represented by $\Delta n = \max(n) - \min(n)$ is plotted versus t_1/V_1 and B/V_1. (b) is similar to (a) but for the VBS with $\Delta\chi = \max(\chi\downarrow) - \min(\chi\downarrow)$ connecting two red sites as shown in (d) where red and blue dots/lines encode the values of n/χ schematically. The phase diagram is shown in (c), including a charge disorder wave (CDW) phase, a fermi liquid (FL) phase and a CDW-VBS coexistent phase.

VII：Lower temperature data

we performed the electronic transport measurement at lower temperature, down to 500 mK and 250 mK, and presented the corresponding experimental results in **Fig. S7(a)** & **S7(b)**. A number of R-T curves spanning a range of *D* [from -0.297 V/nm to -0.324 V/nm] show clear linear tendency (**Fig. S7(c)**), with the boundaries very close to those in higher base temperature. Thereby, the strange metal behaviors persist to much lower temperatures without noticeable shrink of *D* window. In fact, the boundaries of the intermediate phase at zero *T* are determined not by investigating the evolution of the boundaries with temperatures, but by applying standard scaling analysis to determine the quantum critical points, as illustrated below with details.

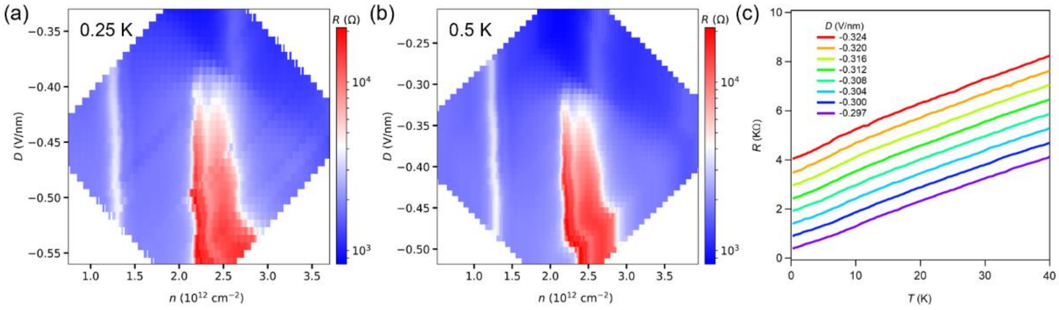

Figure S7: 2D plot of resistance as a function of carrier density n and displacement field D for temperature at (a) 0.25K and (b) 0.5K. (c) Several linear *R*-*T* curves spanning a finite D ranging from -0.324V/nm to -0.297 V/nm.

VIII: Transport Data and theoretical analysis for positive D

The AB-BA stacked TDBG system has a C_{2y} symmetry without vertical *D* field, which means that the systems with opposite *D* fields can be exactly mapped to each other by C_{2y} operation. Therefore, the Wigner crystal state observed for negative *D* field should also appear for positive *D*, and the nature of the resistive states at positive *D* should be of the same nature as those at negative *D*.

We First calculate the band structures of TDBG with positive *D* field (+0.4V/nm) and compare it with the case of negative *D* field (-0.4V/nm), with results shown in **Fig. S8**. We can see that the band structures at *D*=+/- 0.4V/nm are exactly mapped to each other with swapping the two valleys, in which the solid/dashed lines represent the energy bands from the K/K' valley. So, the wavefunctions and band energies are exactly related to each other by the symmetry operation C_{2y} for opposite *D* field.

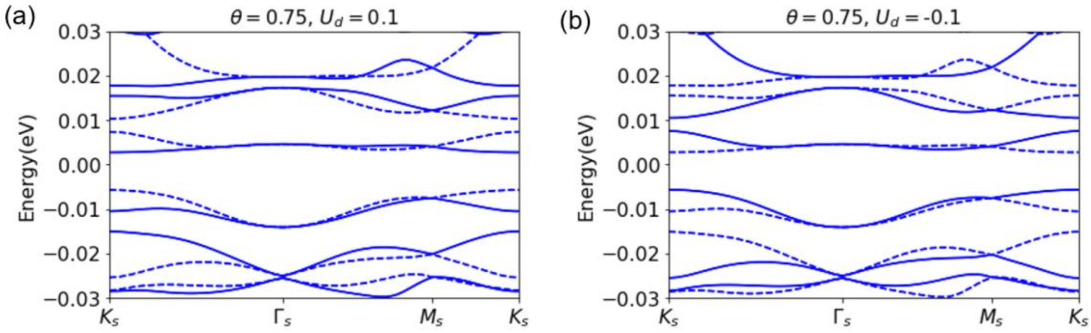

Figure S8: Band structures of AB-BA stacked TDBG at θ=0.75. (a) D=-0.4V/nm, (b) D= 0.4V/nm, where the solid and dashed lines denote the bands from K and K' valleys.

We also carried out additional experiment and present the measured data at positive *D* in **Fig. S9**. We clearly see a displacement field dependent two prominent resistance peaks, at the fillings of $7n_0$ and $(7+2/3)n_0$ (as illustrated in **Fig. S9(b)**). These fillings of the resistance peaks are the same as those peaks present at negative *D*. Moreover, the magnetic field dependence of the resistance at those peaks (**Fig. S9(c), (d)**) is consistent with our observations for negative *D* in the manuscript.

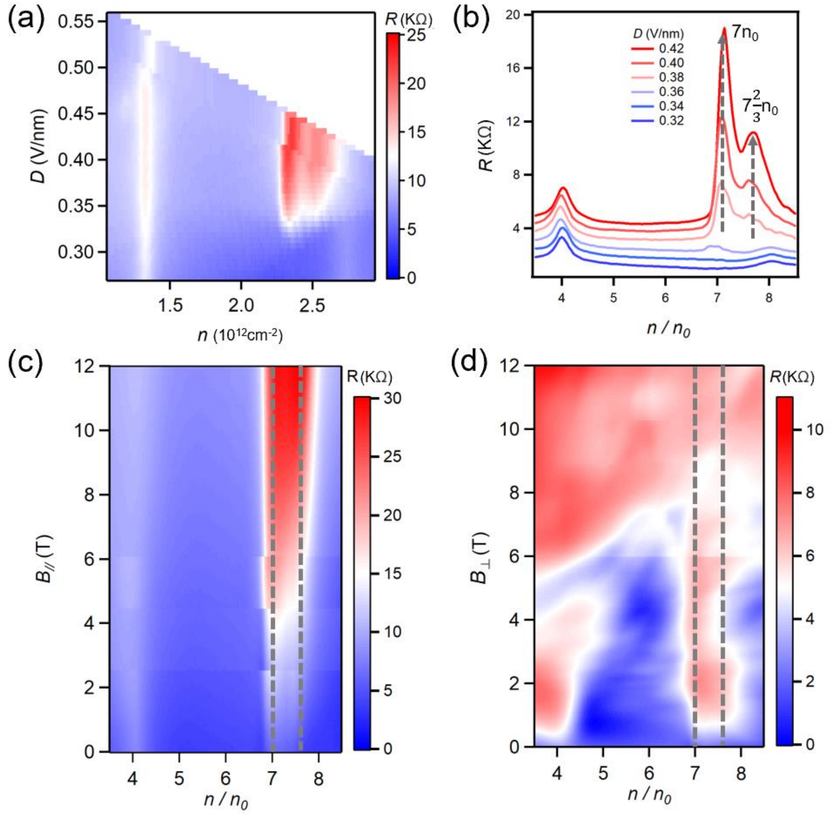

Figure S9: The characterization of the Wigner crystals at positive *D*. (a) The 2D plot of R as a function of n and D. (b) Line plots extracted from (a) for selected displacement fields. (c), (d) 2D map of R as a function of the filling factor and parallel (perpendicular) magnetic field measured at *D* = 0.36 V/nm.

IX. More device for generalized Wigner crystal

Repeating the Wigner crystal state at n/n_0 =7+2/3 should be of crucial importance for substantiating our claims. We indeed have carried out additional measurement on a new device (see photo image in the inset of **Fig. S10(a)**) to repeat the observation of such Wigner crystal states at the same filling. The mapping of R as a function of n and D, as shown in **Fig. S10(a),** showing a *D*-field dependent resistance peak at the filling of 7+2/3. To better demonstrate the fillings of this peak, we plot the line trace of R vs n at D=-0.55V/nm, as shown in **Fig. S10(b)**. We also show a data in our previous device in manuscript in the inset of **Fig. S10(b)**. In addition, we also observe temperature dependence of resistance following ES-type variable range hopping, as well as the *D*-field dependent *dV/dI* spectrum. These key characteristics in the new device with a twisted angle of 0.7 are consistent with the data represented in the original manuscript (a twisted angle of 0.75). This is a strong indication that n/n_0 =7+2/3 filling is quite robust despite the variation of angle.

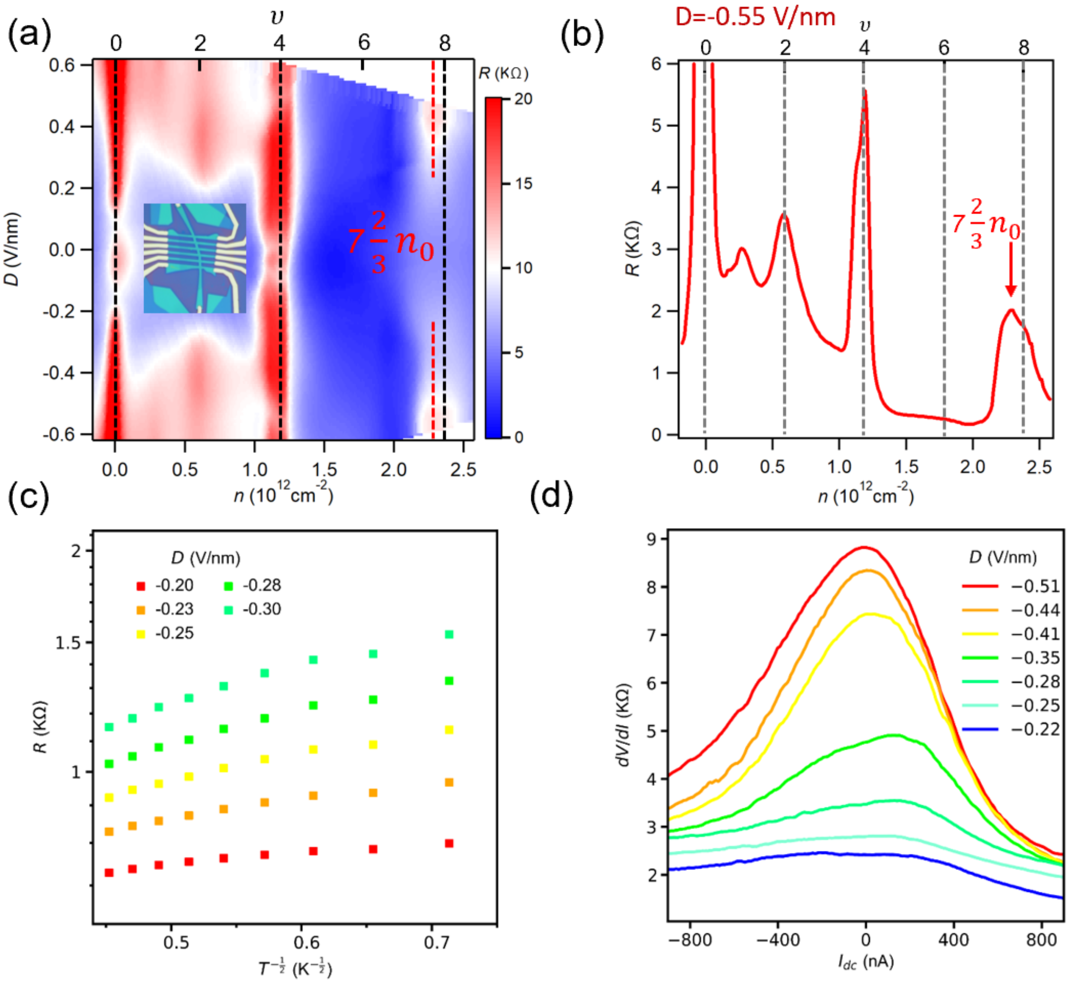

Figure S10: (a) The resistance as a function of n and D, clearly showing a peak a 7+2/3 n0. Inset: optical image another device. (b) Line trace of the resistance as a function of n at D=-0.55V nm. (c) the temperature dependence of log(R) vs $T^{-1/2}$. (d) d*V*/d*I* peaks at different D fields.

X. Estimation of the angle inhomogeneity

We first quantifying the angle inhomogeneity. We performed additional analyses and estimated that the upper bound of the angle inhomogeneity in our device is smaller than $\pm 0.021°$. To make this estimation, we have used the peak width at half height of the moiré peaks. As shown in **Fig. S11**, the moiré peak at fillings of - 4 ranges from 1.245 to 1.395 $\times 10^{12}$ cm^{-2}, and leads to angle ranging from 0.732° ~ 0.774°, which gives rise to $\pm 0.021°$ as the angle inhomogeneity. Since different kinds of disorders including charge impurities, strain distributions and angle inhomogeneity could contribute to the broadening of the moiré peak, the practical angle inhomogeneity in our sample used in the experiment should be much smaller than 0.021°.

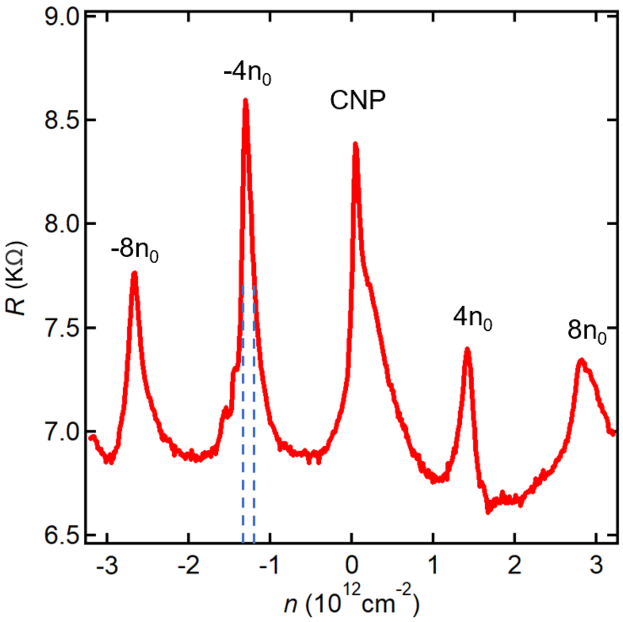

Figure S11. The resistance as a function of carrier density. The dashed lines indicate the half height of the moiré peaks.

We also fabricated a "control" device with significant angle inhomogeneity, as shown in **Fig. S12**. Such angle inhomogeneity leads to significant broadening of moiré gaps at both $n/n_0 = 4$ and 8 filling, and exhibit multiple peaks at the fillings near those moiré gaps. Remarkable, both the broadened moiré peak and other unexpected extra peaks follow the same D-field dependence roughly. In contrast, the moiré peaks in our device are not broadened, and the peak resistances at the filling of $n/n_0 = 7$ and 7+2/3 in our origin device do not follow the same D-field dependence as the $n/n_0 = 8$ filling. In addition, we do not observe extra peak near the fillings of 4 in our origin device, which should appear in the case of angle inhomogeneity. By comparing our origin device to a

“control” device, we can conclude that these experimental observations in our origin device are not consistent with the case of angle homogeneity induced extra peaks.

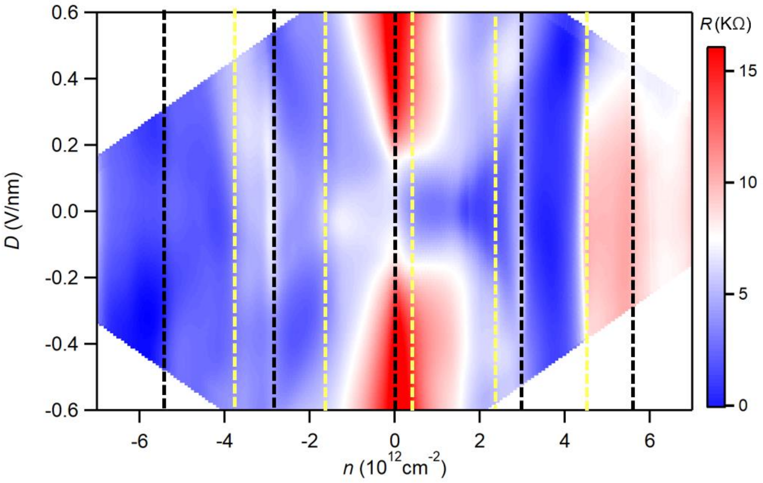

Figure S12. The transport properties of the “control” device. Significant broadening of the moiré gaps (indicated by the dashed lines) and extra peaks that follow the same *D*-dependence are observed.

XI. Estimation of Joule heating

The Joule heating effect can be neglected in our observation of nonlinear *I*-*V* curve in Fig. 2c-d. To address this concern, we measured d*V*/d*I* in moiré gap insulating state on a new device and presented the comparison of nonlinear *I*-*V* curves between our Wigner crystal and single-particle insulating in **Fig. S13**. Compared to the sharp d*V*/d*I* peak observed in our Wigner crystal state, the *dV/dI* peak in moiré gap states is very broad, indicating that the physical origin for the nonlinear *I*-*V* curve in our Wigner crystal should be different from that of the moiré band insulator. With the single-particle moiré gap resistance similar as that of our Wigner crystal (as shown in **Fig. S 13**), the Joule heating should generate similar effect on these two resistive states. However, we note that the d*V*/d*I* curve in single-particle moiré gap state (at $4n_0$ filling) is basically retained unchanged，indicating that Joule heating effect can be neglect for the moiré gap state, and similarly for the Wigner crystal, and thus would not contribute to the observation of nonlinear *I*-*V* curve in Wigner crystal.

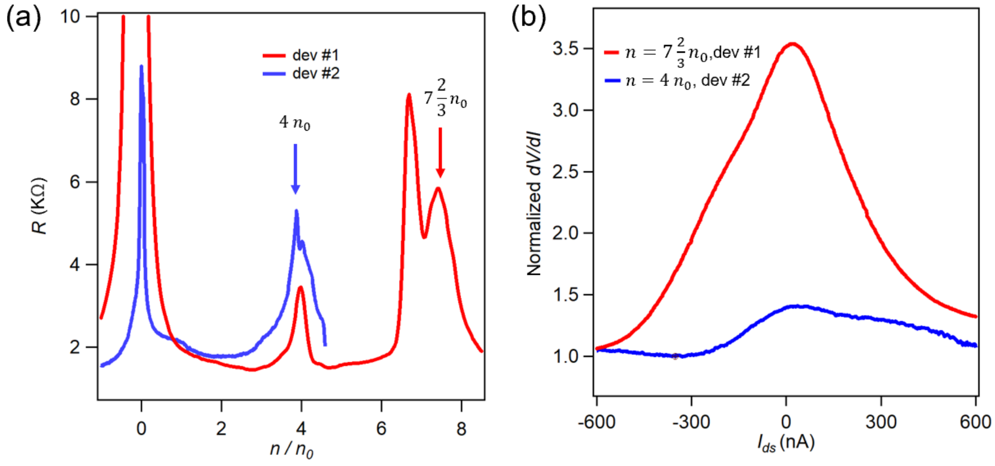

Figure S13. (a) The resistance as a function of carrier density in $4n_0$ moiré gap state and 7+2/3 n_0 Wigner crystal state, showing similar resistance. (b) d*V*/d*I* for these two insulating states. The d*V*/d*I* are normalized according the value.

XII: Calculations of more fermi surface and r_s

We have calculated the Fermi surface and r_s (correlation strength) at fillings of 7+1/3, 3+2/3 that may stabilize the Wigner crystals, with result shown in **Fig. S14** and **S15**. Our calculations show that r_s values (~25 and ~20) in these fillings are smaller than the critical values (r_s=30) required for stabilizing the Wigner crystal. By contrast, r_s ~ 35 can be obtained at the filling of 7+2/3. In addition to this condition, other two conditions *(i.e.* (a) a relatively simple Fermi surface structure; (b) zero Chern number of the flat band) should be satisfied. According to our theoretical calculations, the filling of 7+2/3 in our device possess only one pocket at the gamma point for each valley (**Fig. S16**) and zero Chern numbers, satisfying these criterions, which explains the reason why the Wigner crystals are stabilized at this filling.

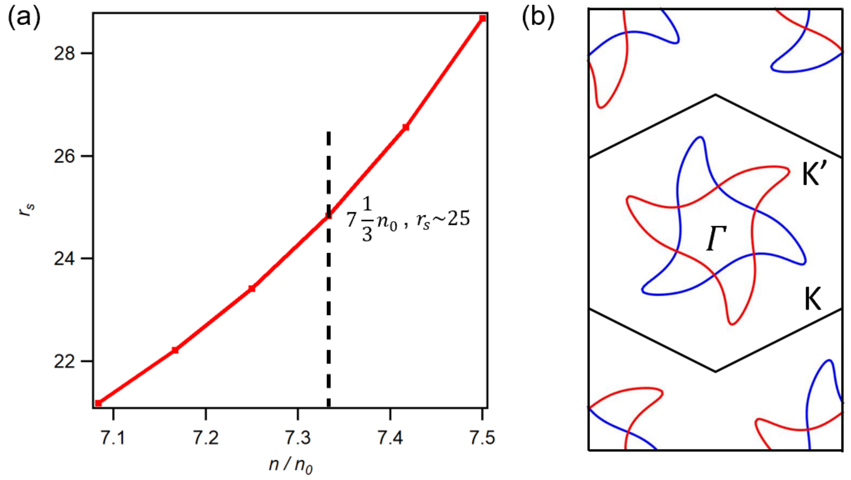

Figure S14: (a) The filling dependence of the r_s value at D=-0.4V/nm around 7+1/3 filling, showing r_s~25 (assuming dielectric constant of BN is 4), which is significantly below the critical value for the Wigner crystal state. (b) The Fermi surface of AB-BA stacked TDBG at θ=0.75 degrees, D=- 0.4V/nm, and at 7+1/3 filling, where the blue and red lines denote the Fermi surfaces at K and K' valleys, respectively.

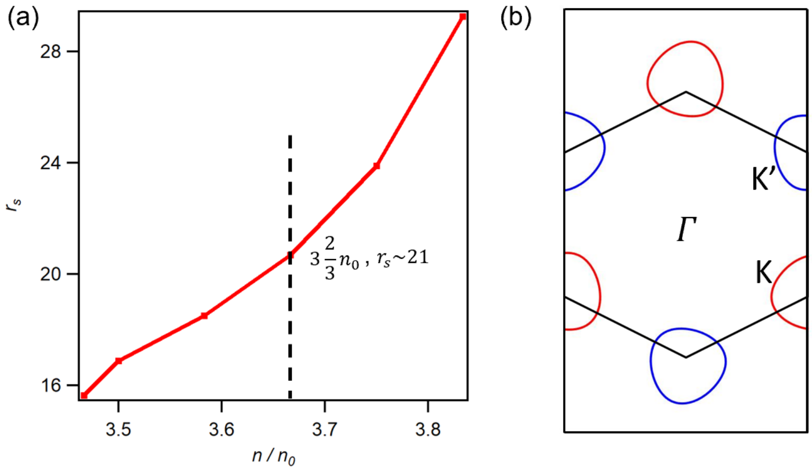

Figure S15: (a) The filling dependence of the r_s value at D=-0.4V/nm around 2+2/3 fillings, showing r_s value at 3+2/3 filling is about 20, far below critical value for the Wigner crystal state. (b) The Fermi surface of AB-BA stacked TDBG at θ=0.75 degrees, D= -0.4V/nm, and at 3+2/3 filling, where the blue and red lines denote the Fermi surfaces at K and K' valleys, respectively.

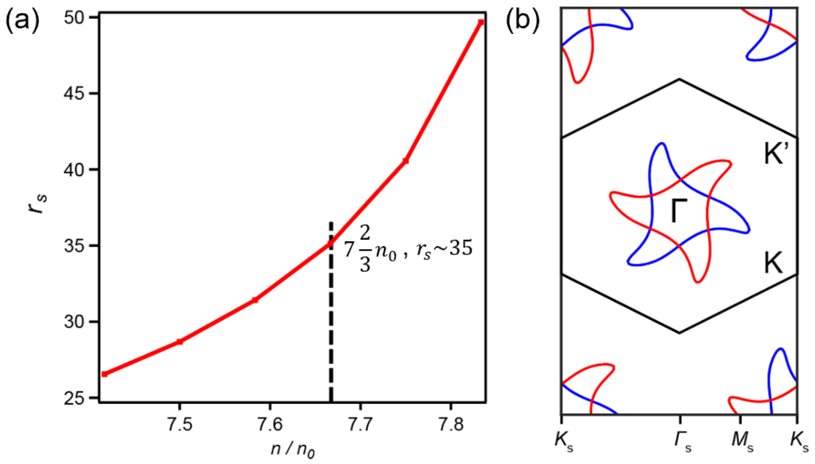

Figure S16: r_s and Fermi surface at n=7+2/3 n_0 at D = -0.4 V/nm, showing a large r_s and simple Fermi surface for stabilizing Wigner crystals.

XIII: The comparison of the resistance at moiré gap and Wigner crystal

The nearby single-particle moiré gap in the proximity to n/n_0=7 and 7+2/3 filling corresponds to n/n_0=8. Based on our experimental results (**Fig. S17**), the state at this filling behaves like metal. To offer insight into why the correlated states at n/n_0=7 and 7+2/3 have higher resistance values than the state at n/n_0=8, we have calculated the indirect gap between the second moiré flat band and the band above it and obtained an indirect gap as small as 0.3 meV. Different from such single-particle moiré gap, the states at n/n_0=7 and 7+2/3 originate from strong electronic correlation. The R-T curves of these states at n/n_0=7 and 7+2/3 follow variable range hopping mechanism ($R \sim T^{-1}$) at low temperature. Although the Arrhenius equation ($R \sim T^{-1}$)) cannot be used to extract the band gap for states at n/n_0=7 and 7+2/3, we can still estimate the "energy gap" by calculating the coulomb interaction between the nearby pinned electron in the moiré lattice, i.e., $U \approx e^2/(4\pi\varepsilon L)$ with L (~30 nm) the lattice constant of the tripled moiré superlattice (similar estimation method has been adopted in ***Nature*** 579, 359 (2020)). The estimated gap is about ~12 meV and is much larger than the indirect band gap at the filling of $8n_0$, leading to a much larger resistance value than that at n/n_0=8.

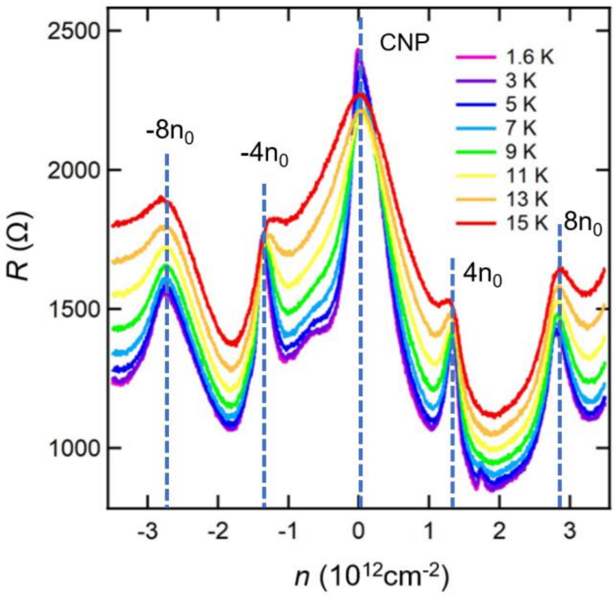

Figure S17: The temperature dependence of *R* vs *n*, showing that the resistance peaks at moiré fillings are all metallic.

XIV: Extraction of gaps at Wigner crystals

For electronic correlation insulating states at n/n_0=7 and 7+2/3, the Arrhenius equation (*R*~*T*$^{-1}$) conventionally employed for extracting single-particle band gap cannot be utilized to extract the energy gap at low temperature. By following the reviewer's request, we adopt the Arrhenius equation to extract the band gap at high temperature, which approaches the thermal melting critical temperature. The extracted gaps are plotted as a function of the displacement field in **Fig. S18**. These values are not the energy scale of the coulomb gaps at low temperature and are valid only at the temperature region where the Winger crystal starts to thermally melt.

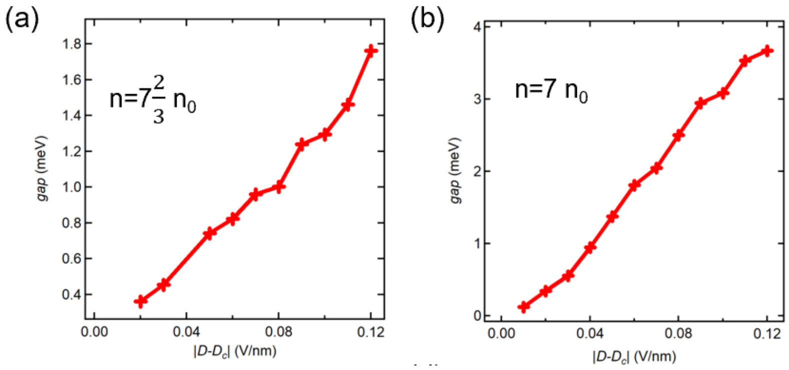

Figure S18: the extracted gap as a function of |D-D_c| at (a) (7+2/3)n_0 and 7n_0.

XV. Ruling out other mechanisms

It is necessary to add more discussions on other possible explanation for the metal-insulator transition. However, both our experimental data on the *D*-field dependent d*V*/d*I* peak, scaling analysis of quantum critical behaviours and our mean-field theory well support that the electronic correlations is the dominant mechanism for metal-insulator transition observed in the Wigner crystal. Such transition cannot be explained by activation across multiple bands within the single-particle framework as the referee proposed. To justify this point, we theoretically estimate the band gap of the moiré gap at filling of 8 and obtain an indirect band gap as small as 0.3 meV. Thermal activation across such small gap to realize metal-insulator transition requires a temperature much lower than 15 K, which corresponds to thermal melting temperature of Wigner crystal in our experiment.

We no rule out the possibility that our observations come from the effect of disorders. Although both the moiré lattice and the disorders can pin the electrons and thus lead to variable range hopping at low temperature, but their pinning effects have different origins. For the moiré lattice, the pinning of electron is mainly stabilized by the strong electron-electron interactions. While for disorder, the coulomb interactions between electrons and charge impurities mainly leads to the pinning of electron. These two distinct mechanisms of the pinning effect give rise to quite different behaviors as listed below, which help us to discriminate our insulating states at fillings of 7 and 7+2/3 from the disorder pinned insulator:

(1) In the disordered electron systems, the disorders will dominate the electric transport and lead to insulating state at low carrier density. Increasing the carrier density would transform the insulating state to metallic state, as shown in the literature (e.g., ***Nature Materials*** 12, 815 (2013)). However, in our measurements, the insulating states only appear at fillings of 7 and 7+2/3, and it vanishes with reducing the carrier density, which is inconsistent with the feature of disorder pinned electronic insulator but coincides with the scenario that the electrons are pinned by the moiré lattice. Moreover, with the carrier density keeping unchanged, applying the displacement field facilitates the metal-insulator transition in our measurement, which again indicates that the insulating states at the filling of 7 and 7+2/3 originate from electronic correlation.

(2) In our measurement, the insulating states will evolve to a strange metal phase with Planckian dissipation limit when the temperature is raised. Such strange metal phase indicates that the electric transport in our device is dominated by the strong electron correlation rather than the disorders suggested by the reviewer.

(3) The extremely high mobility (>10^4 cm^2V^{-1} s^{-1}) accessible in our device indicates that the concentration of disorder is extremely low, excluding the possibility that the disorders dominate the properties of electric transport at low temperature.

Moreover, we can exclude the phonon origin of T-linear resistance.

We performed calculations on the Bloch–Grüneisen temperature, and can safely exclude the possibility that the linear *T* resistance originates from phonons. In the case of phonon-scattering mechanism, the linear-*T* resistance behavior would cross over to a T^4 dependence at temperatures lower than the Bloch–Grüneisen temperature: T_{BG}= $2\hbar v_s k_F/k_B$, where v_s is the sound velocity of the acoustic phonon, k_F is the Fermi wavevector, $\hbar$ is the Planck constant and k_B is the Boltzmann constant [***Phys. Rev. Lett.*** 105, 256805 (2010)]. Taking v_s=2×10^6 cm/s as the sound velocity of graphene, k_F~2.9×10^7 m^{-1} at 7+2/3 filling for AB-BA stacked TDBG with *D*= -0.4V/nm, we obtained T_{BG} = 16 K. Notably, the strange metal behavior persists to the base temperature (250 mK) in our experiment, which is far below the Bloch–Grüneisen temperature calculated above (16 K).
